# The authority of courage and compassion: Healthcare policy leadership in addressing the kidney disease public health epidemic

**DOI:** 10.1111/sdi.12849

**Published:** 2020-01-09

**Authors:** Franklin W. Maddux

**Affiliations:** ^1^ Fresenius Medical Care Bad Homburg Germany

## Abstract

Recent developments in US kidney‐related healthcare policy have made chronic kidney disease (CKD) a societal focus in the United States. In the biggest policy change since the 1972 Social Security Amendments that extended Medicare coverage to patients with kidney failure regardless of age, a 2019 presidential executive order pledged to reduce end‐stage kidney disease, slow CKD progression, increase kidney transplants, and focus on home dialysis care. This manuscript seeks to outline key factors that can enable this milestone moment to evolve a policy framework that improves the health of society while being economically sustainable. Understanding the sociohistorical context of healthcare policy and the related lessons learned demonstrates that policy must take a broader view of the societal and system wide factors that affect chronic illness. Addressing the full breadth of the CKD epidemic requires looking at factors from both inside and outside traditional medical‐pathophysiological environments, including social determinants of health. This more fulsome insight will enable policy to better align the broad range of people and organizations who are working to combat the disease. By creating patient‐centered policy that both evolves with the speed of innovation and addresses root causes of CKD instead of narrowly focusing on symptoms or comorbidities alone, leaders in the public square have an historic opportunity to thoughtfully create the common ground of a lasting policy legacy that improves society's health today and for generations to come.

## 1 INTRODUCTION

How can leaders affect healthcare policy that is sustainable while improving society at large? At its heart, the question policy makers have wrestled with for the better part of the last century is what society will tolerate in terms of preventable illness and death, balanced against our collective tolerance for the expense. Against the backdrop of new policy milestones and ongoing debate, including the merits of socialized vs market‐driven medicine, many grapple with this very question and the way policies have enabled, stimulated, and occasionally derailed the evolution of our delivery system in caring for people with chronic illness.

Chronic kidney disease (CKD) has come of age in the US. In July 2019, the Trump Administration issued an executive order pledging to reduce end‐stage kidney disease (ESKD) by 25% over the next 11 years by improving care quality and slowing disease progression including doubling the number of kidney transplants in the United States. The administration also committed to move dialysis patients away from commercial centers to anticipated less expensive, more convenient in‐home care, with a goal of 80% of patients with incident ESKD to receive a kidney transplant or to obtain their renal replacement therapy at home.

In a press briefing, Joe Grogan, head of the White House Domestic Policy Council, described the set of initiatives as the singular biggest change in kidney care since the passage of the 1972 Social Security Amendments, which created the Medicare ESKD benefit, extending Medicare coverage to kidney failure patients, regardless of age. In light of this historic event, the most striking observation is the 47‐year time gap between these two seminal pieces of healthcare policy and the scientific and market innovations that meanwhile happened in between.

This perspectives manuscript is an attempt to address the question surrounding kidney care‐related healthcare policy in the United States, first looking at the evolution of our health system generally, and then specifically at how a focused approach can enable a path forward to lasting, effective policy.

## 2 HEALTHCARE POLICY IN HISTORIC CONTEXT: ENABLING ACCESS TO INNOVATION

Lags in time between the point a new, socially valuable therapy is available to the time policy can make it widely accepted and utilized by all in need must be thought of as an opportunity cost: the longer it takes for policy changes to bring innovations that address societal health needs to market, the greater the health burden to society, and the greater the economic burden to the system. Because healthcare innovation is an expensive investment in the future good for society, advancements such as scientific breakthroughs, new therapies and health products most often come from the private sector first, requiring policy to evolve—and in many cases catch up—in order to make such innovations available at scale.

The first clinically effective dialysis machine, developed by Dr Willem Johann Kolff in the 1940s and the development of the Scribner Shunt in the 1960s are early examples of technology changing what was a uniformly fatal condition into a chronic, manageable illness. This is also an example of medical technology challenging our social mores, the societal norms of morality that often underpin legal and regulatory changes impacting healthcare. In the US people with kidney failure are uniquely positioned compared to people with other expensive, chronic conditions in that they are eligible for Medicare coverage regardless of age. This anomaly in our Medicare coverage rules is the result of a courageous few on the front lines of kidney care to effectively position the issue with policy makers.

**Figure 1:**
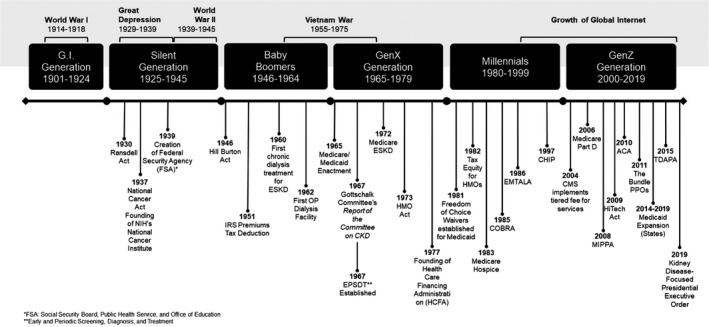
Timeline of US healthcare system policy milestones; kidney care milestones; key societal events; and related generations. (See Supplemental Information of Policy Milestones)

A US healthcare policy and societal journey timeline shows the relationships of policy to society's evolving needs. Health policy from the 1930s through the 1990s focused generally on a piecemeal approach to expanding access to healthcare. The impetus for expanding access was driven in part by the pace of technological advancement in this period, which created a societal need for access to new treatments (Figure 1).

Much of the expansion policy through the late 60s and early 70s was driven by the GI Generation who were at the peak of their policy‐making power. Having lived through the great depression and World War II, the GI Generation was characterized by a sense of community; they supported the New Deal and joined labor unions, and in their later years formed the American Association of Retired Persons, the advocacy organization otherwise known as AARP.^1^ Early policy focused heavily on preparedness and ensuring a healthy workforce. Later, as the GI Generation faced the age of retirement, they were largely responsible for the passage of Medicare.

The experiences in World War II gave rise to new legislative efforts to expand access to healthcare by more specifically addressing the costs of care, which, for the first time, began to act as a barrier. Many policy makers at this time lived through or fought in World War I and II, and it is no surprise that legislation passed in this period focused on ensuring citizens were healthy enough to serve, should the need arise.

At the height of the GI Generation's power in 1965, Congress passed the Medicare and Medicaid benefit, which followed similar societal sentiments toward expanding access to care, this time with a focus on older Americans who could not get access to health insurance through an employer. The creation of the Medicare benefit also set the stage for the 1972 expansion to include coverage for patients with ESKD.

The societal needs of the GI Generation are reflected in legislation ensuring that the Government has some role in financing healthcare. Their legislative achievements also solidified the public‐private approach to coverage that, given the boom in technological advances in the coming years, would ensure that the basic question of the government's role in financing access—and at what cost—would be addressed over and over.

Later, shifts in generational priorities from adult‐focused (preparedness and a healthy workforce) to child‐focused policies (protecting vulnerable people in our population) would leave kidney disease coverage policies largely ignored and incremental for nearly 50 years following the authorization for the ESKD benefit that created a defined system for universal access to a lifesaving therapy. During that those nearly five decades, the benefit cost swelled well beyond estimates. To address costs, on February 12, 1982, the Department of Health and Human Services proposed a change in the law enacted as part of the 1981 Omnibus Reconciliation Act, which would require dialysis services to be reimbursed under a dual prospective composite rate system where one rate per treatment would be set per facility with the same amount paid for dialysis whether performed in‐center or in the patient's home.^2^ While the final rule was enacted in 1983, the policy, and the rates it set, did not keep pace with economics and innovation: while costs were stable or down on a constant currency basis, reimbursement rates did not increase commensurate with inflation; did not account for the costs of introducing new innovations to market; and did not account for the cost related to addressing the entirety of the CKD spectrum instead of just focusing on one point—the end stage—of a complex disease.

The 2010 introduction of the Patient Protection and Affordable Care Act (ACA) would stimulate value‐based care models (such as the End Stage Renal Disease Seamless Care Organizations [ESCO] shared‐savings model, or ultimately the 21st Century Cures Act, which will lead to more access to the Medicare Advantage premium based models) to enable providers and healthcare organizations to reconsider how to address the full CKD continuum, not just kidney failure. The focus went beyond a sole, singular therapy, to a broader view of patient outcomes and experiences in living with kidney failure. It was in the period leading up to the 2011 introduction of the ESKD Prospective Payment System (PPS)—the so called “bundle”—that it became increasingly clear that the population served by the 1972 legislation had led to a small number of people with a life‐threatening disease consuming a disproportionate amount of federal health dollars compared to the number of beneficiaries cared for over the years. This led to greater focus and scrutiny of how to design a better delivery system.

As an example, the Transitional Drug Add‐On Payment Adjustment (TDAPA) implemented on January 1, 2018, required calcimimetics to move from coverage under the Medicare Part D drug coverage system to the Part B medical care services delivery component of the ESKD PPS for injectable, intravenous, and oral forms of the drug class. While this legislation created a pathway for drug classes to find their way into the ESKD bundled payment model, the policy came with distinct operational issues from the start:

1. Transitional Drug Add‐On Payment Adjustment did not account for many logistical requirements needed for providers to operationalize the policy in the field
2. The policy did not address system wide structural issues, such as the dramatic difference in pharmacy dispensing authority between a retail or mail order pharmacy dispensing laws vs those dispensing needs under TDAPA of a dialysis clinic.
3. The policy did not take into account the various logistical pathways patients get medications across the board such as Medicare vs Medicaid vs private insurance.

## 3 CREATING ECONOMICALLY SUSTAINABLE HEALTHCARE POLICY

To be lasting and effective, healthcare policy must broaden its general view of a disease state to include societal and system wide factors that affect the disease. This includes deficiencies in the health system, high rates of unhealthy behaviors and adverse social and environmental conditions (also known as social determinants of healthcare outcomes), leading to poor clinical results.

The narrow focus of the 1972 Medicare policy solely on ESKD and not the full CKD spectrum had three distinct deficiencies: (a) it did not address root cause‐solutions for factors that would contribute to the rise of CKD as a population health epidemic, and instead narrowly focused on one singular part of a much broader health problem; (b) it did not allow for a true understanding of the actual costs involved and the total economic impact of the disease's full spectrum; and (c) it did not address factors contributing to skyrocketing CKD growth and related population health implications, or keep pace with new innovations and emerging science in the field.

Consider for example new gene therapies being developed to treat everything from cancer to blindness. Most are focused on rare disorders with specific genetic subtypes; however, in 2017 a new gene editing‐based therapy was used to effectively cure Janelle Stephenson, a 22‐year‐old woman, of sickle cell disease, one of the more common genetic disorders. As new therapies become available to treat or cure previously debilitating conditions, policy makers will continue to struggle with the very question legislators faced in 1972. What is the role of the government in ensuring access to these technologies, and at what cost?

The urgency and intensity of the question has escalated in recent years as healthcare costs continue to increase exponentially and more Americans feel the strain of healthcare consuming nearly 18% of the country's gross domestic product.^3^ Policy makers today do not have the luxury of considering just one expensive lifesaving technology. Interestingly, 47 years after the passage of the ESKD Medicare benefit—still the only disease‐specific coverage mandate—regulators and kidney advocates find themselves asking whether the very existence of the benefit might constrain the development of new technological advances.

In 2017, a study conducted by the University of Washington in conjunction with the Bill and Melinda Gates Foundation quantified the impact of over 300 major diseases and injuries around the world, showing obesity as “a growing and disturbing global public health crisis.”^4^ This *Global Burden of Disease* study stated that 2.2 billion—or 30% of the world's population—are either overweight or obese, leading to burgeoning global health problems and morbidity.^5^

**Figure 2:**
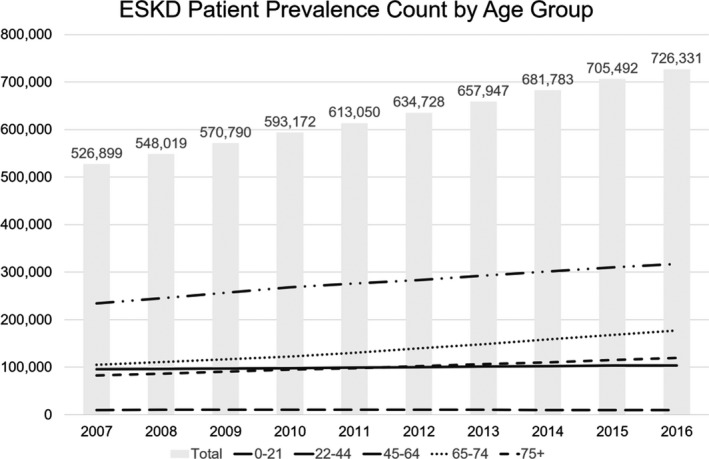
Kidney disease on the rise: US snapshot^6^

At 13%, the United States reported the highest percentage of obese children and young adults. Since 1980, the study showed the frequency of obesity has doubled in more than 70 countries. The two leading causes of death were identified as cardiovascular disease and diabetes, with CKD ranked as the second‐leading cause of disability. The US is a microcosm of this global epidemic with more than 30 million Americans diagnosed with kidney disease, a growing number the healthcare system must prepare to adequately support (Figure 2).

In the United States alone, Medicare annually spends $114 billion in managing all aspects of kidney disease, more than 20% of all Medicare spending.^6^ The numbers on a global scale are expected to increase substantially over the next decade.

In his recounting of the origins of the Medicare ESKD benefit, Richard Rettig wrote that “[…] widespread publicity of lives lost for lack of scarce medical resources was necessary, including specific dramatization of identified lives at stake. Finally, the number of patients being kept alive had to increase to the point where they simply could not be ignored.”^4^ While the ESKD benefit was briefly considered as part of a broader effort to provide more robust coverage of catastrophic illness, ultimately policy makers arrived at a narrower, ESKD‐focused benefit that was a balance of saving lives and costs to the system.^4^

This societal choice was driven in part by the development of kidney care device and medical therapeutic innovations that shaped societal mores toward the government's responsibility to make a particular therapy available to those who could benefit but could not otherwise afford it. While there has been much debate and some consternation over the evolution of this therapy since 1972, we must not forget that the alternative for patients was assured death. On the passage of the ESKD benefit, Senator Russell Long later recalled:

As chairman [of the Senate Finance Committee], I sat there and thought to myself: We are the greatest nation on earth, the wealthiest per capita. Are we so hard pressed that we cannot pay for this? A life could be extended 10 to 15 years. You're not going to make any money that way. But it struck me as a case of compelling need.^7^

What policy makers in 1972 perhaps could not fully understand was that the dialysis machine was just the tip of the iceberg, and they could not anticipate the breakneck pace of technological innovation in healthcare in the coming decades.^8^ While many of these technologies are lifesaving or life altering, they come with a hefty price tag.

Constrained by low reimbursement set each year by the Centers for Medicare and Medicaid Services, advances in treatment for ESKD have been slow and incremental relative to disease states such as cancer. Medicare reimbursement for dialysis services is chronically underfunded despite the large aggregate spending.^9^ In comparison, government incentives in, for example, new treatments for rare diseases, has led to a surge in new treatment options over the last 35 years.^10^

To address this disparity, the Department of Health and Human Services (HHS), in partnership with the American Society of Nephrology, launched in 2019 the Kidney Innovation Accelerator, which seeks to establish a public‐private innovation fund to foster breakthroughs in treatments for kidney disease including renal replacement therapies. In a blog post, HHS Chief Technology Officer Bruce Greenstein wrote, “[T]he American taxpayer is billed annually $35 billion for a decades old technology with mortality rates higher than most cancers […]”.^11^

The true environment today is that structural issues in the current policy framework require that organizations use cross‐payer or subsidies to maintain sustainability of the dialysis therapy systems much as acute care hospitals use service line subsidies to support unsustainable therapies they must provide to the communities they serve. This reality is due to policy framework forecasting that underestimated, many years ago, the scope and breadth of the number of people and conditions that need treatment when trying to manage a complex ESKD population.

The reality is that commercial patients subsidize patients covered by Medicare and Medicaid in many cases and these structural issues lead to discord for many stakeholders in the system about how to best care for the whole population of people with ESKD equitably. It is the patients’ desire (along with their nephrologists and other caregivers) to survive and function at the highest level possible in their communities that will pressure policy makers to resolve these structural problems and create a resilient, reliable healthcare system with enough efficiency to sustain the broad access to high quality care required to help people with kidney disease lead productive lives.

Distinct structural solutions will be further required to avoid unintended consequences (as seen in the TDAPA extension), which will further confuse the generation of an accurate cost baseline when time comes to rebase the prospective payment system for ESKD care in the United States. As policy makers continue to seek the right balance of access and cost, perhaps the structure and funding of the Medicare ESKD benefit can serve as a lesson: policy solutions should seek to avoid the slowing of future investment. TDAPA is ongoing today and there is the potential for serial transitions from one drug to another within a class as they enter the market. CMS has not clarified its intent and is still unclear whether the first drug that enters the market, or each new drug that enters the market in a new functional category, will get the 2 years add‐on payment. The Hypoxia Inducible Factor prolyl hydroxylase inhibitors (HIF‐PHIs, also known as HIF stabilizers) will potentially set a precedent for this particular issue if they are deemed part of the future bundle and placed into the TDAPA portfolio.

## 4 PATIENT‐CENTERED POLICY: DRIVER OF EFFICIENCY, BETTER OUTCOMES, AND SYSTEM WIDE ALIGNMENT

The evolution toward patient‐centered health policy provides the basis for how the policy maker must respond.

Three hypothetical patient profiles demonstrate the need for policy that is *relevant* to the needs of individual patients and their unique circumstances, underscoring the critical importance for policy to enhance *power and choice* for patients:

- The low income, elderly ESKD patient with multiple comorbidities may live in a “food desert” and not have adequate access to food that is critical for her nutritional competence.
- The patient who has an acute illness leading to acute kidney injury has a substantial chance to recover kidney function but will also require care coordination during recovery while completing the therapeutic course required for resolution of the acute illness. This requires flexibility in the system to keep the patient in the correct site of care that avoids attenuation of recovery while maintaining every chance for the patient to ultimately return to a normal life without severe chronic kidney impairment.
- Thirdly, the 40‐year‐old patient who is destined to have a kidney disorder because of known genetic risks wants to find the right medical team to help him navigate the complex decisions around modality and timing of various kidney replacement therapies while staying independent enough to work and manage a family.

These examples represent both a patient's universal will to survive and the complexity in which the policy framework must accommodate many different individual life scenarios. Policy will fall short without addressing the issues associated with avoidable health crises in kidney failure patients and the need for insights and actions that help these individuals remain productive.

For many patients, the toll of chronic illness, and the impacts their home environment and other external factors have on their care are substantial. These environmental impacts or “social determinants of health” greatly influence a patient's ultimate care outcomes and utilization of available health resources to address their medical problems. If we think about the integration of these social determinants of health with the medical outcomes of individuals, the interrelationship between social spending and medical spending becomes apparent very quickly.

**Figure 3:**
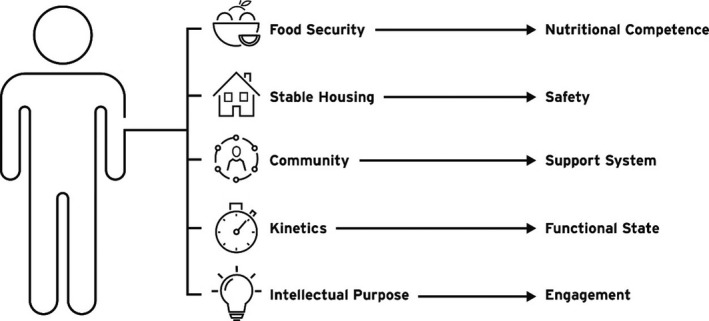
Successful and sustainable healthcare policy must address kidney disease‐related social determinants of healthcare as additional drivers of care outcomes

Five social themes that are beginning to reflect in policy decisions affecting people with kidney disease include food security, stable housing, community, kinetics, and intellectual purpose. These five determinants are each connected to a clinical result and influence how well patients can succeed in life while living with their kidney disease (Figure 3).

Kidney care policy that supports larger investment in science and innovation is required to evolve the field toward preventing CKD progression, addressing people with earlier stages of kidney disease and reducing the burden of people moving toward ultimate organ failure.

Creating a patient‐centered policy process requires an environment focused on delivering precise, personalized care. Individual patients should get the right therapy at the right time for their individual circumstance of kidney failure, but within a structured framework of options. This requires policy to adapt to our evolving understanding of the disease state, and to align policy with breaking market innovations that have promise to make therapies more precise.

For example, forward‐thinking policy that considers the molecular and genetic levels of a disease will offer greater opportunity to create a case for more precise therapies. This may involve shifting our fundamental approach to how we approach kidney diseases: our system based on a pathologic classification of diseases leaves minimal opportunity to subclass a disorder by molecular or genetic signatures.

Many research facilities have begun to untangle the morass of molecular variations of clinical kidney disorders currently stratified into major visual pathological classifications such as “Minimal Change Disease,” “Focal Segmental Glomerulosclerosis,” and “Diabetic Nephropathy.” At the end of the day, almost all kidney diseases relate to the loss of either glomerular function or a tubulointerstitial insult that results in destruction of tissue and scarring of the kidney.

If we can imagine a time when kidney diseases have their pathologic classification enhanced by molecular markers and genetic variations, we can also imagine new opportunities to improve outcomes and lower costs by delivering targeted therapies to which a specific, individual patient is more likely to respond.

**Figure 4:**
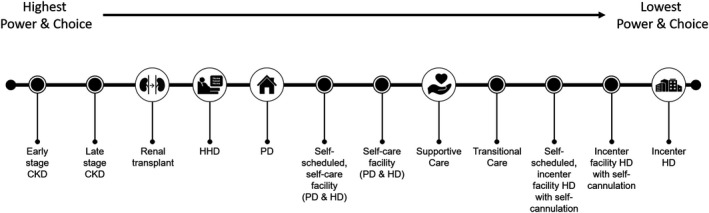
Patient power and choice relative to different care modalities across the patient care journey

Policy that enables more precise personalized kidney care must also have a full view of both the patients’ lifetime care journey and the role of patient power and choice in their care (Figure 4).

## 5 POLICY AS LASTING LEGACY: FINDING A COMMON GROUND

The phrase “I care about people” is the sentiment at the heart of healthcare policy that is truly focused on a *societal need.* In fact, the very starting point of healthcare policy lies in its intent. This manuscript is written on the foundation of three basic assumptions: (a) the fundamental intent of healthcare policy is to improve society for the greater good; (b) policy which deviates from this societal‐focused intent is inherently flawed in that it would be impossible to align the motivations of people, organizations, and institutions responsible for enacting such tangential policy; and (c) healthcare policy at its core focuses on fundamental needs of society and addresses the universal human experience of a shared will to survive.

In their 2018 paper on evaluating evidence behind US policy mandates in dialysis care, Erickson and Winkelmayer compared two federal mandates affecting the care of ESKD patients receiving hemodialysis, specifically the 2004 reform of the physician Monthly Capitation Payment (MCP) for nephrologists providing outpatient dialysis care and the inclusion of injectable medications into the ESRD bundle for dialysis patients in 2011.

The analysis indicated how differences in the clarity of stated policy objectives, the quality of data collected at the time of policy enactment, and prior evidence supporting policy objectives can influence a policy's success as well as efforts to evaluate its success. Furthermore, it showed how clarity of purpose and intent can help avoid unintended consequences such as the misalignment of the very people and organizations responsible for enacting policy through incentives not clearly defined.^12^ In short, policy without the mutual understanding created by a foundational common ground may be policy doomed to fail.

**Figure 5:**
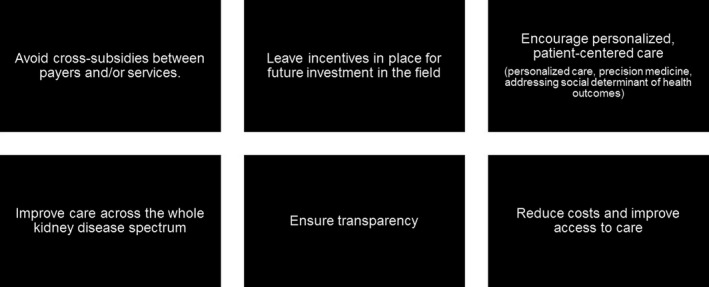
Areas of focus for future policy

To summarize what history has taught, future policy should consider distinct imperatives in order to be socially and economically viable (Figure 5). With the magnitude of the economic, societal, medical, and logistical understandings required for truly effective policy, it is perhaps the compassionate policy maker who is best equipped to create lasting legislation.

For a compassionate policy maker, nothing human is alien: no human condition, no disease, no way of living, and no way of dying. This compassion is not only courage, but also true authority because it considers the full societal implications of healthcare policy decisions and does not tolerate the political pressures so fleeting in incumbency. The compassionate policy maker does not get distracted by the special interests of enterprising cliques; instead, through courage, the compassionate policy maker finds common ground by breaking through barriers between languages and cultures, rich and poor, educated and illiterate, creating a lasting policy legacy for the greater good of a society, both today and tomorrow.
