# Supplementary material for: The authority of courage and compassion: Healthcare policy leadership in addressing the kidney disease public health epidemic
Source: Semin Dial. 2020 Jan 9;33(1):35–42. doi: 10.1111/sdi.12849 (PMC7004129; doi:10.1111/sdi.12849)
Supplement: Supplementary file 1 [file SDI-33-35-s001.pdf]

Glossary of Policy Milestones

The **Ransdell Act** enacted May 26, 1930 reorganized, expanded and re-designated the *Laboratory of Hygiene* (created in 1887) as the National Institute of Health.

The National Cancer Institute (NCI) was established through the **National Cancer Act of 1937**, signed into law by President Franklin D. Roosevelt. Its passage represented the culmination of nearly three decades of efforts to formalize the U.S. government’s place in cancer research.

The **Federal Security Agency** (FSA) was an independent agency of the United States government established in 1939 that oversaw food and drug safety as well as education funding and the administration of public health programs.

The Hospital Survey and Construction Act (**Hill Burton Act**) called for the construction of hospitals and related health care facilities and was designed to provide federal grants and guaranteed loans to improve the physical plant of the nation's hospital system with the requirement that such facilities could not discriminate based on race, color, national origin, or creed and must provide a modicum of free care.

The **premium tax credit** (**PTC**) is a refundable tax credit in the United States.

Early and Periodic Screening, Diagnostic and Treatment (EPSDT) is the child health component of Medicaid.

In 1967, **Gottschalk chaired a U.S. government committee** that recommended government support for kidney transplants and artificial kidney machines for patients with kidney failure.

The Health Maintenance Organization Act, informally known as the **federal HMO Act**, is a federal law that provides for a trial federal program to promote and encourage the development of health maintenance organizations (HMOs).

In 1977, the Health Care Financing Administration (**HCFA**) was established under the Department of Health, education and Welfare (HEW). HCFA became responsible for the coordination of Medicare and Medicaid.

Freedom of Choice Waivers are generally used to enroll certain beneficiaries into mandatory managed care for Medicaid

The **Tax Equity and Fiscal Responsibility Act of 1982**, under rules implemented in April 1985, creates incentives for HMOs and other competitive medical plans to significantly expand their participation in Medicare on an at-risk basis.

COBRA is a federal law that may allow you to temporarily keep health coverage after your employment ends

The Emergency Medical Treatment and Labor Act (**EMTALA**) is a federal law that requires anyone coming to an emergency department to be stabilized and treated, regardless of their insurance status or ability to pay.

Children's Health Insurance Program (**CHIP**) is an insurance program that provides low-cost health coverage to children in families that earn too much money to qualify for Medicaid but not enough to buy private insurance.

Medicare did not cover outpatient prescription drugs until January 1, 2006, when it implemented the Medicare Part D prescription drug benefit, authorized by Congress under the "Medicare Prescription Drug, Improvement, and Modernization Act of 2003." This Act is generally known as the "**MMA**."

The Medicare Improvements for Patients and Providers Act (**MIPPA**) of 2008 is a multi-faceted piece of legislation related to Medicare.

The Health Information Technology for Economic and Clinical Health Act (**HITECH**) is part of the American Recovery and Reinvestment Act (ARRA) of 2009 and creates incentives related to health care information technology, including incentives for the use of electronic health record (EHR) systems among providers.

Patient Protection and Affordable Care Act. ... (It's sometimes known as “PPACA,” “**ACA**,” or “Obamacare.”) The law provides numerous rights and protections that make health coverage more fair and easy to understand, along with subsidies (through “premium tax credits” and “cost-sharing reductions”) to make it more affordable.

The ESRD PPS (the **Bundle**) provides a patient-level and facility-level adjusted per treatment (dialysis) payment to ESRD facilities for renal dialysis services provided in an ESRD facility or in a beneficiary’s home.

Medicaid expansion, as allowed under the Affordable Care Act and shaped by a 2012 Supreme Court decision, allows states to broaden who is eligible under the program. It would expand the barriers to include those who are within 138 percent of the federal poverty line, which amounts to nearly $26,000 annually.

The intent of the **TDAPA**, as implemented at 42 CFR 413.234(c), is to facilitate beneficiary access to certain qualifying, new injectable or intravenous products by allowing payment for these drugs and biologicals while the necessary utilization data is collected.

The President’s Executive Order on **Advancing American Kidney Health** is dedicated to the following principles:

(a) prevent kidney failure whenever possible through better diagnosis, treatment, and incentives for preventive care;

(b) increase patient choice through affordable alternative treatments for ESRD by encouraging higher value care, educating patients on treatment alternatives, and encouraging the development of artificial kidneys; and

(c) increase access to kidney transplants by modernizing the organ recovery and transplantation systems and updating outmoded and counterproductive regulations.
